# Adjuvant radiotherapy for patients with clinical T3–4 oral and oropharyngeal cancer who achieved major pathologic response after neoadjuvant immunochemotherapy and surgery: a propensity score-matched retrospective study

**DOI:** 10.3389/fimmu.2025.1681587

**Published:** 2026-01-20

**Authors:** Menghua Li, Shiyan Yang, Lili Liu, Wanming Hu, Shida Yan, Yani Zhang, Mingyuan Du, Xianlu Gao, Chulin Yang, Liji Zheng, Chunyan Chen, Jian Zhou, Jiabin Lu, Ming Song, Shuwei Chen

**Affiliations:** 1 Department of Head and Neck Surgery, Sun Yat-sen University Cancer Center, Guangzhou, China; 2 State Key Laboratory of Oncology in South China, Guangzhou, China; 3 Guangdong Provincial Clinical Research Center for Cancer, Guangzhou, China; 4 Department of Pathology, Sun Yat-sen University Cancer Center, Guangzhou, China; 5 Department of Radiation Oncology, Sun Yat-sen University Cancer Center, Guangzhou, China; 6 Department of Radiology, Sun Yat-sen University Cancer Center, Guangzhou, China

**Keywords:** adjuvant (chemo)radiotherapy, head and neck cancer, immunotherapy, major pathologic response, neoadjuvant therapy, pathologic complete response

## Abstract

**Background:**

Neoadjuvant chemotherapy combined with immunotherapy results in high pathologic response rates in locally advanced oral and oropharyngeal cancer (OC/OPC). It is unclear if patients with clinical T3-4 (cT3-4) OC/OPC at initial diagnosis can safely omit adjuvant radiotherapy (ART) after significant pathological downstaging.

**Methods:**

This retrospective cohort study included cT3–4 OC/OPC patients who achieved a major pathologic response (MPR) after neoadjuvant immunochemotherapy between July 2019 and May 2024. Patients were categorized by whether they received ART. Propensity score matching was used to balance baseline characteristics. Local recurrence-free survival (LRFS), locoregional recurrence-free survival (LRRFS), distant metastasis-free survival (DMFS), and overall survival (OS) were compared between cohorts.

**Results:**

A total of 247 patients were eligible, with a median follow-up of 31 months (IQR, 20-41). The 2-year survival outcomes were favorable: LRFS 93.4%, LRRFS 85.0%, DMFS 95.6%, and OS 93.0%. In the matched cohorts (74 pairs), ART significantly improved 2-year LRFS (100% vs. 85.5%, *p* = 0.001), and LRRFS (91.5% vs. 77.5%, *p* = 0.014), but not DMFS (96.4% vs. 95.6%, *p* = 0.740), and OS (96.5% vs. 90.0%, *p* = 0.093). These benefits remained significant among patients with ypT0–2 tumors after matching.

**Conclusions:**

Omitting ART in patients with cT3–4 OC/OPC who achieve MPR after neoadjuvant immunochemotherapy and surgery significantly compromises oncological outcomes. Further investigation is necessary to optimize adaptive de-escalation strategies for this population.

## Introduction

About two-thirds of patients with head and neck squamous cell carcinoma (HNSCC) have locally advanced disease, requiring multimodal management (1, 2). Adjuvant radiotherapy (ART) reduces the risk of locoregional recurrence, distant metastasis, and mortality in patients with locally advanced oral and oropharyngeal squamous cell carcinoma (OC/OPC) after radical surgical resection (3). These benefits are most pronounced in patients with two or more regional lymph nodes (LNs) involved, extracapsular spread of disease, positive surgical margins, perineural invasion (PNI), lymphovascular invasion (LVI), and T3–4 disease (4–6).

Neoadjuvant therapy reduces tumor burden and may enable organ preservation. Prospective clinical trials have shown that neoadjuvant chemotherapy, particularly when combined with immunotherapy, yields favorable efficacy (7–9). The potential benefits include less extensive surgery and improved survival outcomes for a subset of patients exhibiting a favorable response (10, 11). Major pathologic response (MPR) is a surrogate marker for satisfactory survival and may facilitate treatment de-escalation to improve quality of life (12–15).

With the increasing use of neoadjuvant immunochemotherapy, many patients with initial cT3–4 OC/OPC may achieve significant pathologic downstaging. Currently, no prospective data support the benefits of ART for these patients, leading to clinical uncertainty and variability in practice. This study retrospectively analyzes the benefits of ART for cT3–4 OC/OPC patients who achieved MPR following neoadjuvant immunochemotherapy and surgery. The purpose of this study is to evaluate whether ART to the head or neck can be safely omitted.

## Patients and methods

### Study population and eligibility criteria

We retrospectively collected clinicopathologic data from patients with cT3–4 OC/OPC treated with neoadjuvant immunochemotherapy followed by radical surgery at Sun Yat-sen University Cancer Center from July 2019 to May 2024. This study was approved by the hospital’s Institutional Review Board (approval number B2025-644-01), and the ethics committee review specifically waived the need for informed consent.

Inclusion criteria included: (1) patients with cT3-4a OC/OPC according to the eighth edition of American Joint Committee on Cancer (AJCC), (2) at least two cycles of neoadjuvant immunochemotherapy before surgery, (3) surgical resection with curative intent, and (4) MPR (≤ 10% residual viable tumor cells) of primary tumor.

Exclusion criteria included: (1) concurrent distant metastasis at diagnosis, (2) positive surgical margins, and (3) follow up period less than 12 months.

### Data collection

Clinical and pathologic data were collected from the institutional HNSCC database. Pretreatment staging was assessed using high-resolution MRI, CT, and neck ultrasound. The chemotherapy regimen comprised albumin-bound paclitaxel (260 mg/m²) combined with either cisplatin (60 mg/m²) or lobaplatin (30 mg/m²), administered in three-week per cycle. Immunotherapy consisted of anti-PD-1 agents, including pembrolizumab (200 mg per cycle), nivolumab (3 mg/kg per cycle), tislelizumab (200 mg per cycle), camrelizumab (200 mg per cycle), sintilimab (200 mg per cycle), and toripalimab (240 mg per cycle).

Pathologic tumor staging and response evaluation were conducted by two certified pathologists. The response to neoadjuvant therapy was assessed using the resected tumor specimen, with residual viable tumor cells evaluated via H&E staining of all slides. The pathologists were blinded to patient groupings.

ART was delivered using intensity modulated radiotherapy techniques. Doses were 60–66 Gy in 28–33 fractions for the high-risk clinical target volume (CTV) and 54–56 Gy in 28–33 fractions for the low-risk CTV.

### Statistical analysis

Nonparametric data were analyzed with the Wilcoxon rank-sum test, while categorical data were summarized by frequency and compared using the chi-square test for proportions. The outcomes of interest included local recurrence-free survival (LRFS), locoregional recurrence-free survival (LRRFS), distant metastasis-free survival (DMFS), and overall survival (OS). LRFS was defined as the duration from surgery to the occurrence of primary tumor recurrence. LRRFS was defined as the duration from surgery to the occurrence of primary tumor recurrence or LN metastasis. DMFS was defined as the duration from surgery to the occurrence of distant metastasis. OS was defined as the duration from surgery to death. Follow-up time was calculated from surgery to the last follow-up, hospitalization, or death.

Propensity score 1:1 matching was conducted using logistic regression with a caliper width of 0.02 to balance covariates related to treatment selection, including sex, age, tobacco use, alcohol use, primary tumor site, histologic grade, pathologic response, pathologic T stage (ypT, AJCC eighth), pathologic N stage (ypN, AJCC eighth), PNI, LVI, and lymph node yield (LNY). The Matching package in R (version 4.4.2) was used for matching. Clinical outcomes and the efficacy of ART were analyzed with Kaplan-Meier survival curves and the log-rank test, adjusted for propensity score matching (PSM).

Statistical analyses were conducted using SPSS software (version 27) and R software (version 4.4.2), with a *p* value of < 0.05 considered significant.

## Results

### Patient characteristics

**Figure 1:**
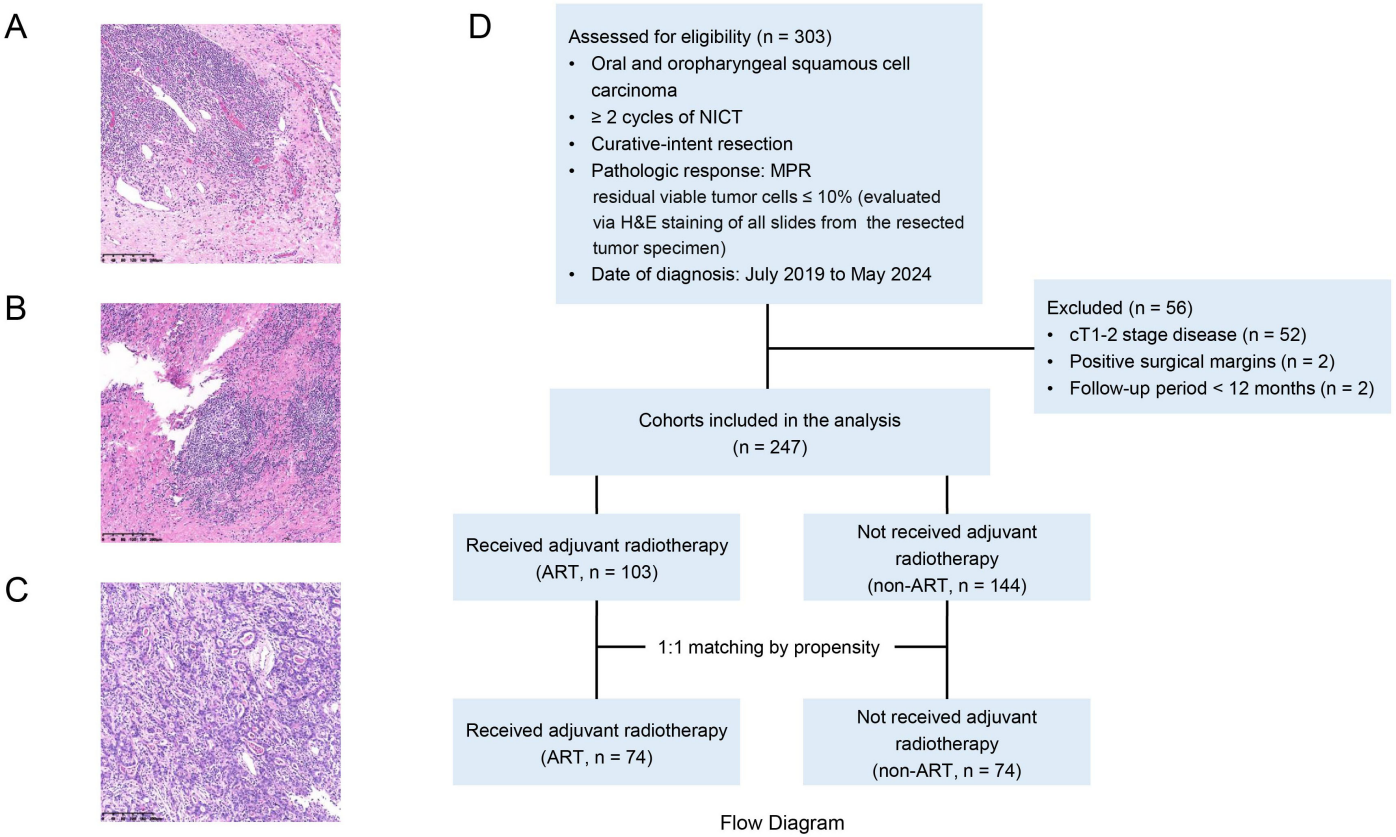
Representative hematoxylin-and-eosin stained slide image of **(A)** complete pathologic response (no viable tumor), **(B)** major pathologic response (5% viable tumor), **(C)** unfavorable pathologic response (40% viable tumor), and **(D)** Flow Diagram.

A total of 247 patients were eligible (**Figure 1**). Their clinicopathologic characteristics are summarized in **Table 1**. The median age at diagnosis was 54 years (IQR, 46-64). Of the patients, 103 (41.7%) received ART while 144 (58.3%) did not (non-ART). Of note, 31 patients (12.6%) in the non-ART cohort received adjuvant anti-PD-1 maintenance therapy, with a median of 8 cycles (range: 5–35). Patients in the ART cohort tended to be younger (*p* = 0.008) and more likely to have LN metastasis (*p* < 0.001). No significant differences were found between cohorts in the distribution of sex, tobacco use, alcohol use, primary tumor site, histologic grade, number of neoadjuvant immunochemotherapy cycles, pathologic response, ypT stage, PNI, and LNY (*p*>0.05). After PSM, characteristics were balanced (*p* > 0.05), with 74 patients in each cohort.

**Table 1:** Clinicopathologic characteristics according to adjuvant radiotherapy.

Characteristic	Before PSM				After PSM			
	Overall, n=247	Non-ART, n=144	ART, n=103	*p*	Overall, n=148	Non-ART, n=74	ART, n=74	*p*
Sex, No. (%)				0.739				0.834
Male	202 (81.8)	119 (82.6)	83 (80.6)		120 (81.1)	61 (82.4)	59 (79.7)	
Female	45 (18.2)	25 (17.4)	20 (19.4)		28 (18.9)	13 (17.6)	15 (20.3)	
Age, years				0.015				0.82
Median (IQR)	54 (46-64)	57 (48-64)	51 (44-60)		53 (45-60)	53 (45-60)	52 (45-61)	
Tobacco use, No. (%)				0.330				0.718
No	171 (69.2)	96 (66.7)	75 (72.8)		105 (70.9)	51 (68.9)	54 (73.0)	
Yes	76 (30.8)	48 (33.3)	28 (27.2)		43 (29.1)	23 (31.1)	20 (27.0)	
Tumor site, No. (%)				0.739				1
Oral cavity	185 (74.9)	106 (73.6)	79 (76.7)		112 (75.7)	56 (75.7)	56 (75.7)	
p16- Oropharynx	25 (10.1)	16 (11.1)	9 (8.7)		13 (8.8)	6 (8.1)	7 (9.5)	
p16+ Oropharynx	37 (15.0)	22 (15.3)	15 (14.6)		23 (15.5)	12 (16.2)	11 (14.9)	
Histologic grade, No. (%)				0.301				0.503
Poor	57 (23.1)	31 (21.5)	26 (25.2)		33 (22.3)	19 (25.7)	14 (18.9)	
Moderate	112 (45.3)	62 (43.1)	50 (48.5)		76 (51.4)	38 (51.4)	38 (51.4)	
Well	78 (31.6)	51 (35.4)	27 (26.2)		39 (26.4)	17 (23.0)	22 (29.7)	
cT, No. (%)				0.897				0.322
T3	108 (43.7)	62 (43.1)	46 (44.7)		67 (45.3)	30 (40.5)	37 (50.0)	
T4a	139 (56.3)	82 (56.9)	57 (55.3)		81 (54.7)	44 (59.5)	37 (50.0)	
cN, No. (%)				0.001				0.225
N0	119 (48.2)	80 (55.6)	39 (37.9)		79 (53.4)	42 (56.8)	37 (50.0)	
N1	29 (11.7)	21 (14.6)	8 (7.8)		17 (11.5)	10 (13.5)	7 (9.5)	
N2	95 (38.5)	42 (29.2)	53 (51.5)		49 (33.1)	22 (29.7)	27 (36.5)	
N3	4 (1.6)	1 (0.7)	3 (2.9)		3 (2.0)	0 (0)	3 (4.1)	
ypT, No. (%)				0.284				1
T0	168 (68.0)	99 (68.8)	69 (67.0)		106 (71.6)	53 (71.6)	53 (71.6)	
T1	56 (22.7)	33 (22.9)	23 (22.3)		32 (21.6)	16 (21.6)	16 (21.6)	
T2	17 (6.9)	11 (7.6)	6 (5.8)		8 (5.4)	4 (5.4)	4 (5.4)	
T3	3 (1.2)	1 (0.7)	2 (1.9)		2 (1.4)	1 (1.4)	1 (1.4)	
T4	3 (1.2)	0 (0.0)	3 (2.9)					
ypN, No. (%)				< 0.001				1
N0	186 (75.3)	127 (88.2)	59 (57.3)		117 (79.1)	59 (79.7)	58 (78.4)	
N1	35 (14.2)	12 (8.3)	23 (22.3)		23 (15.5)	11 (14.9)	12 (16.2)	
N2	24 (9.7)	5 (3.5)	19 (18.4)		8 (5.4)	4 (5.4)	4 (5.4)	
N3	2 (0.8)	0 (0.0)	2 (1.9)					
NICT cycles, No. (%)				0.471				0.499
2 cycles	65 (26.3)	42 (29.2)	23 (22.3)		36 (24.3)	21 (28.4)	15 (20.3)	
3 cycles	158 (64.0)	88 (61.1)	70 (68.0)		92 (62.2)	43 (58.1)	49 (66.2)	
≥4 cycles	24 (9.7)	14 (9.7)	10 (9.7)		20 (13.5)	10 (13.5)	10 (13.5)	
Pathologic response, No. (%)				0.680				1
PCR	169 (68.4)	100 (69.4)	69 (67.0)		106 (71.6)	53 (71.6)	53 (71.6)	
Non-PCR	78 (31.6)	44 (30.6)	34 (33.0)		42 (28.4)	21 (28.4)	21 (28.4)	
PNI, No. (%)				0.164				1
No	242 (98.0)	143 (99.3)	99 (96.1)		147 (99.3)	74 (100)	73 (98.6)	
Yes	5 (2.0)	1 (0.7)	4 (3.9)		1 (0.7)	0 (0)	1 (1.4)	
LVI, No. (%)				0.417				1
No	246 (99.6)	144 (100)	102 (99.0)		148 (100)	74 (100)	74 (100)	
Yes	1 (0.4)	0 (0.0)	1 (1.0)		0	0	0	
LNY, No. (%)				0.216				1
≤ 18	78 (31.6)	50 (34.7)	28 (27.2)		53 (35.8)	27 (36.5)	26 (35.1)	
> 18	169 (68.4)	94 (65.3)	75 (72.8)		95 (64.2)	47 (63.5)	48 (64.9)	
Adjuvant therapy, No. (%)								
non-ART	144 (58.3)				74 (50.0)			
PD-1 maintenance	31 (12.6)				18 (12.2)			
ART	103 (41.7)				74 (50.0)			

ART, adjuvant radiotherapy; PCR, pathologic complete response; PNI, perineural invasion; LVI, lymphovascular invasion; LNY, lymph node yield; PSM, propensity score matching; ypT, pathologic T stage; ypN, pathologic N stage.

### Role of ART in the whole cohort and matched cohort

The median follow-up for the whole cohort was 31 months (IQR, 20-41). Fourty-three patients (17.4%) experienced failure events. Local recurrence occurred in 14 patients (5.7%), including 11 in the non-ART cohort and 3 in the ART cohort. Locoregional recurrence was noted in 36 patients (14.6%), including 25 in the non-ART cohort and 11 in the ART cohort. Thirteen patients (5.3%) developed distant metastasis, with 6 in the non-ART cohort and 7 in the ART cohort. The estimated 2-year LRFS, LRRFS, DMFS, and OS for the whole cohort were 93.4%, 85.0%, 95.6%, and 93.0%, respectively.

**Figure 2:**
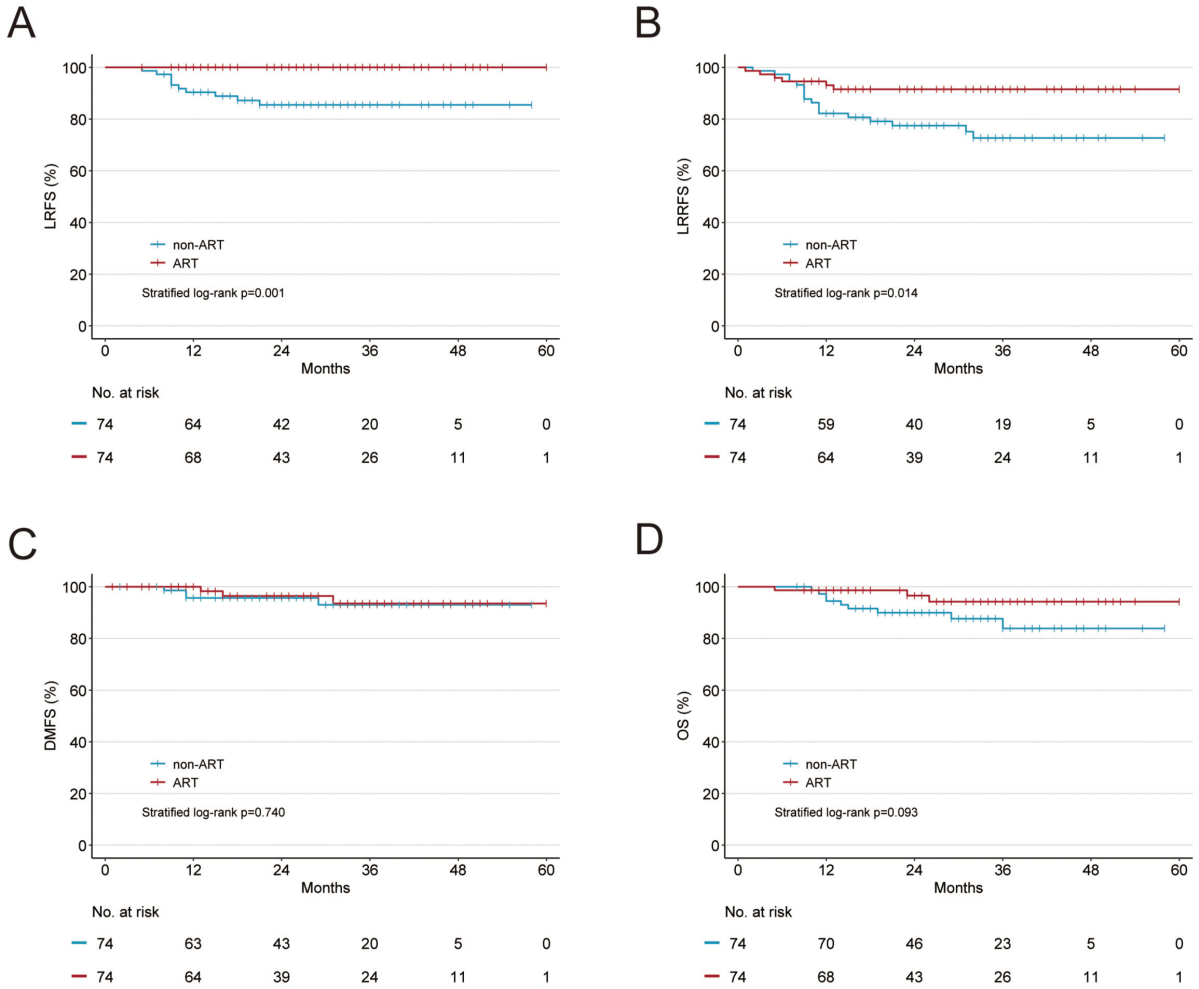
Kaplan-Meier curves of **(A)** local recurrence-free survival (LRFS), **(B)** locoregional recurrence-free survival (LRRFS), **(C)** distant metastasis-free survival (DMFS), and **(D)** overall survival (OS) in the propensity score–matched cohort.

After adjusting for PSM, the median follow-up for the ART and non-ART cohorts were 30.5 months (IQR, 21-40) and 31.5 months (IQR, 16-42), respectively. In the non-ART cohort, 10 patients (13.5%) experienced local recurrence, 18 patients (24.3%) had locoregional recurrence, and 4 patients (5.4%) developed distant metastasis. In contrast, the ART cohort had 0 patients with local recurrence, 6 patients (8.1%) with locoregional recurrence, and 3 patients (4.1%) with distant metastasis. ART was associated with statistically significant improvements in LRFS by 14.5%, LRRFS by 14.0%, but not in DMFS and OS. The survival outcomes in the ART and non-ART cohorts were 100% *versus* 85.5% for LRFS (*p* = 0.001; **Figure 2A**), 91.5% *versus* 77.5% for LRRFS (*p* = 0.014; **Figure 2B**), 96.4% *versus* 95.6% for DMFS (*p* = 0.740; **Figure 2C**), and 96.5% *versus* 90% (*p* = 0.093; **Figure 2D**), respectively.

**Figure 3:**
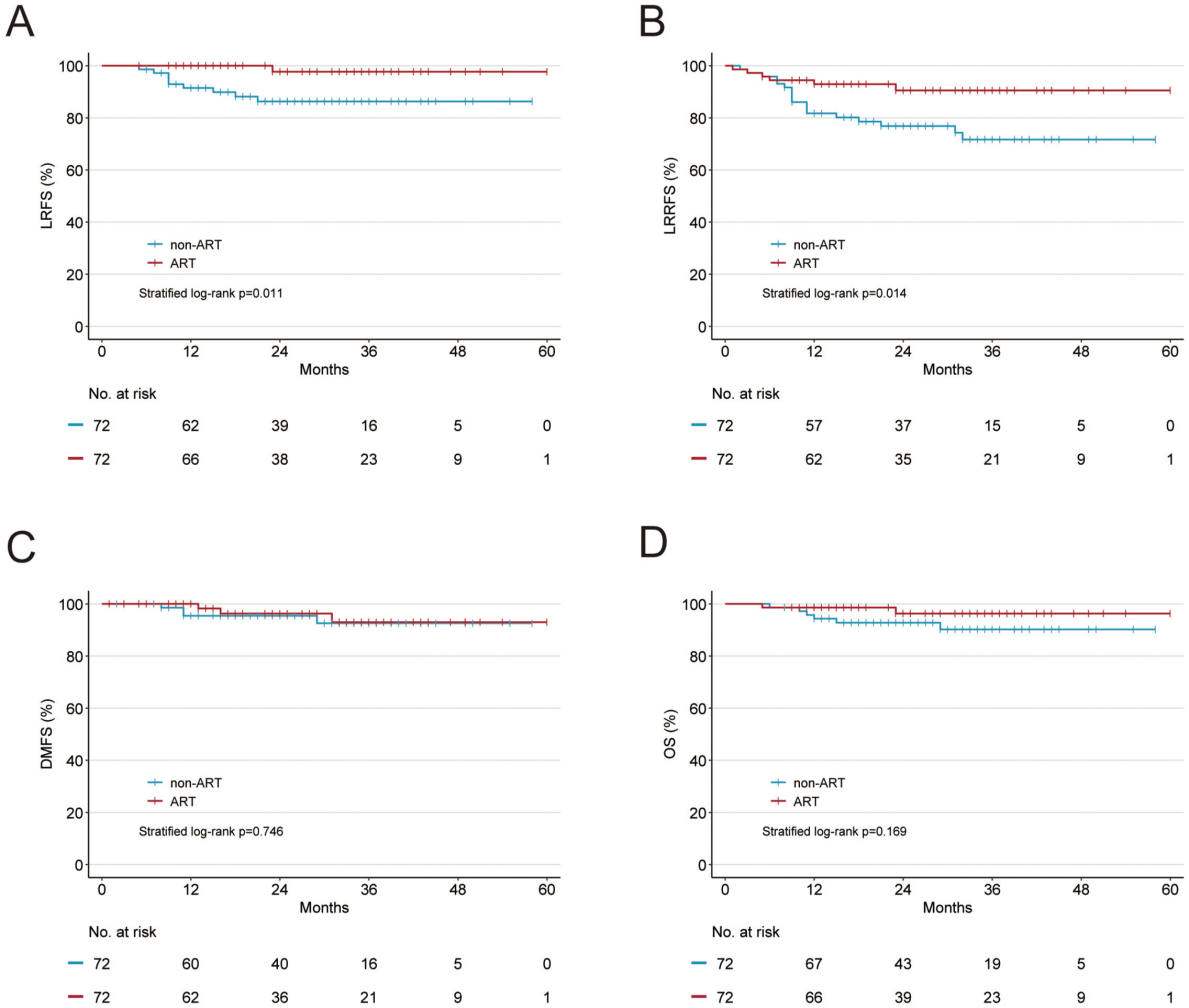
Kaplan-Meier curves of **(A)** local recurrence-free survival (LRFS), **(B)** locoregional recurrence-free survival (LRRFS), **(C)** distant metastasis-free survival (DMFS), and **(D)** overall survival (OS) in patients with ypT0–2 tumor after propensity score–matching.

Given that pT3–4 disease constitutes a high-risk factor for radiotherapy, we subsequently performed an analysis excluding patients with ypT3–4 disease from the entire cohort. After PSM, each cohort comprised 72 patients. The baseline characteristics are presented in **Supplementary Table S1**. ART continued to demonstrate significant improvements in LRFS (97.7% vs. 86.3%, *p* = 0.011; **Figure 3A**), LRRFS (90.5% vs. 76.9%, *p* = 0.014; **Figure 3B**), but not in DMFS (96.3% vs. 95.5%, *p =* 0.746; **Figure 3C**), and in OS (96.3% vs. 92.8%, *p* = 0.169; **Figure 3D**). The detailed survival outcomes are presented in **Table 2**.

**Table 2:** Two-year survival rates with and without adjuvant radiotherapy.

Variable	Non-ART	ART	*P*
Whole cohort			
2-year LRFS	91.8	97.4	0.140
2-year LRRFS	83.3	87.4	0.227
2-year DMFS	96.7	93.8	0.240
2-year OS	92.7	93.4	0.963
PSM cohort			
2-year LRFS	85.5	100	0.001
2-year LRRFS	77.5	91.5	0.014
2-year DMFS	95.6	96.4	0.740
2-year OS	90	96.5	0.093
ypT0–2 PSM cohort			
2-year LRFS	86.3	97.7	0.011
2-year LRRFS	76.9	90.5	0.014
2-year DMFS	95.5	96.3	0.746
2-year OS	92.8	96.3	0.169

ART, adjuvant radiotherapy.

### Adverse events

Of the 103 patients who received ART, 25 (24.3%) experienced late adverse effects of grade 3 or higher, including one death due to lung complications (**Table 3**). Four had grade 4 effects (skin, mucosa, bone, hematologic), while 20 patients (19.4%) had grade 3 effects, primarily in mucosa (7 cases), pharynx/esophagus (4 cases), skin (3 cases), subcutaneous tissue (2 cases), salivary gland (2 cases), bone (1 case), and other neurologic (1 case).

**Table 3:** Number of patients having late toxicity who received adjuvant radiotherapy.

Late adverse effect	ART (n = 103)		
	Grade 3	Grade 4	Grade 5
Skin	3	1	0
Mucous membrane	7	1	0
Subcutaneous tissue	2	0	0
Salivary gland	2	0	0
Pharynx/esophagus	4	0	0
Larynx	0	0	0
Lung	0	0	1
Spinal cord	0	0	0
Bone	1	1	0
Joint	0	0	0
Brain	0	0	0
Other neurologic	1	0	0
Hematologic	0	1	0
Renal	0	0	0
Total	20 (19.4)	4 (3.9)	1 (1.0)

ART, adjuvant radiotherapy.

## Discussion

It is uncertain if patients with cT3–4 OC/OPC at initial diagnosis can safely omit ART after achieving MPR following neoadjuvant immunochemotherapy. Our study findings demonstrate that omission of ART significantly compromises survival outcomes, leading to reduced LRFS and LRRFS, while DMFS, and OS are not significantly affected. These reductions remain statistically significant among patients with ypT0–2 tumors.

Neoadjuvant therapy is an essential approach in the treatment of many solid tumors. However, two phase 3 trials failed to show a survival benefit of neoadjuvant chemotherapy with TPF regime in HNSCC (16, 17). The addition of immune checkpoint inhibitors to neoadjuvant chemotherapy has shown significant pathologic responses, but survival outcomes are rarely reported. The recent KEYNOTE-689 trial demonstrated that perioperative administration of PD-1 inhibitors significantly improves event-free survival (EFS) in patients with locally advanced HNSCC, increasing the 3-year EFS rate from 46.4% to 57.6% (18). The most notable benefit was observed in reducing distant disease progression, while no significant effect was seen on local disease control. However, the study was unable to distinguish the specific benefits attributable to adjuvant therapy. Additionally, the low pathologic response rate to anti-PD-1 monotherapy limited the potential for further subgroup analyses, particularly concerning MPR. In our cohort of OC/OPC patients who achieved MPR to neoadjuvant therapy, outcomes were satisfying, with a 2-year LRFS of 91.8%, 2-year LRRFS of 83.3%, 2-year DMFS of 96.7%, and 2-year OS of 92.7%. These results are comparable to a study of 26 patients with HNSCC who received ART after neoadjuvant immunochemotherapy and surgery, which reported a 2-year PFS of 85.1% and OS of 89% (19). Our findings indicate that patients exhibiting MPR may have survival benefits, consistent with a prior research (13). Further study is needed to confirm long-term benefits.

The satisfying efficacy of neoadjuvant immunochemotherapy suggests the potienal of treatment de-escalation in the adjuvant setting. One possible approach may be that omit ART for favorable responders. In a pilot study of 23 patients with resectable HNSCC, 11 patients did not receive ART after neoadjuvant therapy. All patients remain disease-free at a median follow-up of 7.3 months, but long-term outcomes are not yet available (20). A study also found that omitting ART after neoadjuvant chemotherapy and surgery for oropharyngeal squamous cell carcinoma led to excellent locoregional control (21). Ju et al. reported no significant differences in OS or LRRFS between patients with and without ART (22). However, a substantial discrepancy was observed in 10-year OS between the ART and non-ART cohorts (58.9% *versus* 44.1%). In contrast, the findings of our study diverge from these results. Specifically, omission of ART was associated with a significant compromise in local and locoregional control. Notably, our data suggest that ART should not be avoided in OC/OPC patients after achieving MPR following neoadjuvant therapy, despite their generally favorable outcomes. This disadvantage may be related to the extent of surgery performed following significant tumor downstaging. Currently, there is no consensus on the appropriate extent of resection following neoadjuvant therapy. In this study, patients underwent extended surgery with resection margins exceeding 1 cm, determined by their response to neoadjuvant immunochemotherapy. Consequently, a considerable proportion of patients might have undergone less extensive surgery if the procedure had been based solely on the initial clinical stage. Although previous studies have reported satisfactory survival outcomes following de-escalated surgery after neoadjuvant therapy (23, 24), our study suggests caution.It indicates that reducing the extent of surgery after significant pathologic downstaging may compromise radical oncologic control and may necessitate ART. Therefore, the optimal extent of surgical resection requires further investigation.

Reducing the extent of ART for favorable responders may constitute a viable strategy. In a phase II trial (NCT05476965), patients who achieved a MPR to neoadjuvant immunochemotherapy and surgery received a de-escalated dose of radiotherapy (less than 54 Gy/25 fractions). Ten patients received low-dose ART, and 12 received full-dose. At a median follow-up of 23 months, there were no relapses in the low-dose group (25). The OPTIMAL trial showed that induction chemotherapy with dose and volume de-escalated definitive radiotherapy for HPV-positive oropharyngeal squamous cell carcinoma leads to favorable oncologic outcomes and reduced toxicity (26). And The OPTIMAL II trial found that single-modality radiotherapy or transoral robotic surgery after a favorable response to neoadjuvant immunochemotherapy resulted in excellent survival and functional outcomes (27). Similarly, the DEPEND trial indicated that de-escalated definitive radiotherapy after favorable responses to neoadjuvant immunochemotherapy in HPV-negative HNSCC improved survival with fewer acute toxic effects (28). The efficacy of reducing the radiotherapy dose or volume in ART for favorable pathologic responders may represent a promising de-escalation treatment.

Outcomes among patients with OC/OPC with favorable pathologic response to neoadjuvant therapy may probably continue to improve due to advancements in systemic therapy, particularly the use of immune checkpoint inhibitors. These developments may allow more patients to transition from advanced to early-stage tumors, making the findings from the present study applicable to more patients in the future.

Our study has several limitations. First, we only reported early oncological outcomes, requiring further long-term follow-up. Second, we did not analyze the impact of concomitant regimens during ART. Finally, as a retrospective study, it may have potential biases.

## Conclusions

Patients with cT3–4 OC/OPC who achieved favorable pathologic response after neoadjuvant immunochemotherapy and surgery benefit from ART. Further research is needed to optimize adaptive de-escalation strategies for this population.

## Data Availability

The data supporting the conclusions of this article are available from the corresponding author upon reasonable request.

## Supplementary material

Supplementary Table 1 Clinicopathologic characteristics in the ypT0–2 cohort. ART, adjuvant radiotherapy; PCR, pathologic complete response; PNI, perineural invasion; LVI, lymphovascular invasion; LNY, lymph node yield; PSM, propensity score matching; ypT, pathologic T stage; ypN, pathologic N stage.

## References

[1] Chow LQM . Head and neck cancer. New Engl J Med. (2020) 382:60–72. doi: 10.1056/NEJMra1715715, PMID: 31893516

[2] National Comprehensive Cancer Network . Head and Neck Cancers (Version 3.2024). (2024). Available online at: https://www.nccn.org/professionals/physician_gls/pdf/head-and-neck.pdf.

[3] Awan M Akakpo KE Shukla M Graboyes EM Pipkorn P Puram SV . The substantial omission of indicated postoperative radiotherapy in patients with advanced-stage oral cancer in the US-A call to action. JAMA Otolaryngology-Head Neck Surg. (2021) 147:907–9. doi: 10.1001/jamaoto.2021.1744, PMID: 34383035 PMC8517743

[4] Bernier J Domenge C Ozsahin M Matuszewska K Lefèbvre JL Greiner RH . Postoperative irradiation with or without concomitant chemotherapy for locally advanced head and neck cancer. New Engl J Med. (2004) 350:1945–52. doi: 10.1056/NEJMoa032641, PMID: 15128894

[5] Cooper JS Pajak TF Forastiere AA Jacobs J Campbell BH Saxman SB . Postoperative concurrent radiotherapy and chemotherapy for high-risk squamous-cell carcinoma of the head and neck. New Engl J Med. (2004) 350:1937–44. doi: 10.1056/NEJMoa032646, PMID: 15128893

[6] Bernier J Cooper JS Pajak TF van Glabbeke M Bourhis J Forastiere A . Defining risk levels in locally advanced head and neck cancers:: A comparative analysis of concurrent postoperative radiation plus chemotherapy trials of the EORTC (22931) and RTOG (9501). Head Neck-Journal Sci Specialties Head Neck. (2005) 27:843–50. doi: 10.1002/hed.20279, PMID: 16161069

[7] Masarwy R Kampel L Horowitz G Gutfeld O Muhanna N . Neoadjuvant PD-1/PD-L1 inhibitors for resectable head and neck cancer A systematic review and meta-analysis. JAMA Otolaryngology-Head Neck Surg. (2021) 147:871–8. doi: 10.1001/jamaoto.2021.2191, PMID: 34473219 PMC8414366

[8] Wise-Draper TM Gulati S Palackdharry S Hinrichs BH Worden FP Old MO . Phase II clinical trial of neoadjuvant and adjuvant pembrolizumab in resectable local-regionally for advanced head and neck squamous cell carcinoma. Clin Cancer Res. (2022) 28:1345–52. doi: 10.1158/1078-0432.CCR-21-3351, PMID: 35338369 PMC8976828

[9] Ferris RL Spanos WC Leidner R Gonçalves A Martens UM Kyi C . Neoadjuvant nivolumab for patients with resectable HPV-positive and HPV-negative squamous cell carcinomas of the head and neck in the CheckMate 358 trial. J ImmunoTherapy Cancer. (2021) 9:e002568. doi: 10.1136/jitc-2021-002568, PMID: 34083421 PMC8183204

[10] Chaukar D Prabash K Rane P Patil VM Thiagarajan S Ghosh-Laskar S . Prospective phase II open-label randomized controlled trial to compare mandibular preservation in upfront surgery with neoadjuvant chemotherapy followed by surgery in operable oral cavity cancer. J Clin Oncol. (2022) 40:272. doi: 10.1200/JCO.21.00179, PMID: 34871101

[11] Silver JA Bouganim N Richardson K Henry M Mascarella MA Ramirez-GarciaLuna J . Quality of life after neoadjuvant chemotherapy and transoral robotic surgery for oropharynx cancer. JAMA Otolaryngology-Head Neck Surg. (2024) 150:65–74. doi: 10.1001/jamaoto.2023.3781, PMID: 38060238 PMC10704343

[12] Ju W-T Liu Y Wang L-Z Li J Ren G-X Sun J . Phase III trial of docetaxel cisplatin 5-fluorouracil induction chemotherapy for resectable oral cancer suggests favorable pathological response as a surrogate endpoint for good therapeutic outcome. Cancer Commun. (2021) 41:279–83. doi: 10.1002/cac2.12136, PMID: 33471949 PMC7968883

[13] Zhong L-P Zhang C-P Ren G-X Guo W William WN Sun J . Randomized phase III trial of induction chemotherapy with docetaxel, cisplatin, and fluorouracil followed by surgery versus up-front surgery in locally advanced resectable oral squamous cell carcinoma. J Clin Oncol. (2013) 31:744–51. doi: 10.1200/JCO.2012.43.8820, PMID: 23129742 PMC5569675

[14] Reijers ILM Menzies AM van Akkooi ACJ Versluis JM van den Heuvel NMJ Saw RPM . Personalized response-directed surgery and adjuvant therapy after neoadjuvant ipilimumab and nivolumab in high-risk stage III melanoma: the PRADO trial. Nat Med. (2022) 28:1178. doi: 10.1038/s41591-022-01851-x, PMID: 35661157

[15] Hellmann MD Chaft JE William WN Jr. Rusch V Pisters KMW Kalhor N . Pathological response after neoadjuvant chemotherapy in resectable non-small-cell lung cancers: proposal for the use of major pathological response as a surrogate endpoint. Lancet Oncol. (2014) 15:E42–50. doi: 10.1016/S1470-2045(13)70334-6, PMID: 24384493 PMC4734624

[16] Haddad R O’Neill A Rabinowits G Tishler R Khuri F Adkins D . Induction chemotherapy followed by concurrent chemoradiotherapy (sequential chemoradiotherapy) versus concurrent chemoradiotherapy alone in locally advanced head and neck cancer (PARADIGM): a randomised phase 3 trial. Lancet Oncol. (2013) 14:257–64. doi: 10.1016/S1470-2045(13)70011-1, PMID: 23414589

[17] Cohen EEW Karrison TG Kocherginsky M Mueller J Egan R Huang CH . Phase III randomized trial of induction chemotherapy in patients with N2 or N3 locally advanced head and neck cancer. J Clin Oncol. (2014) 32:2735. doi: 10.1200/JCO.2013.54.6309, PMID: 25049329 PMC4876357

[18] Uppaluri R Haddad RI Tao YA Le Tourneau C Lee NY Westra W . Neoadjuvant and adjuvant pembrolizumab in locally advanced head and neck cancer. New Engl J Med. (2025) 393:37–50. doi: 10.1056/NEJMoa2415434, PMID: 40532178

[19] Xu Y Wang J Zhang Y Wu R Chen X Huang X . Efficacy of postoperative radiotherapy following induction immunochemotherapy and surgical resection in locally advanced head and neck squamous cell carcinoma. Int J Radiat Oncol Biol Phys. (2024) 120:E802–2. doi: 10.1016/j.ijrobp.2024.07.1762

[20] Dunn L Cracchiolo J Ho AL Sherman EJ Ganly I Ghossein R . Neoadjuvant cemiplimab with platinum-doublet chemotherapy and cetuximab to de-escalate surgery and omit adjuvant radiation in locoregionally advanced head & neck squamous cell carcinoma (HNSCC). Ann Oncol. (2024) 35:S619–9. doi: 10.1016/j.annonc.2024.08.921

[21] Costantino A Sampieri C Sim NS De Virgilio A Kim S-H . Adjuvant radiation sparing after neoadjuvant chemotherapy and TORS in selected HPV-positive oropharyngeal cancer. Laryngoscope. (2025) 135:1401–8. doi: 10.1002/lary.31940, PMID: 39632778

[22] Ju W Zhang Y Liu Y Sun J Li J Dong M . Can adjuvant radiotherapy be omitted for oral cavity cancer patients who received neoadjuvant therapy and surgery? A retrospective cohort study. Int J Surg. (2023) 109:879–86. doi: 10.1097/JS9.0000000000000353, PMID: 36999830 PMC10389426

[23] Cracchiolo JR Starc MT Wong RJ Ho A Ganly I Pfister DG . Defining response-adapted surgery after neoadjuvant therapy in oral cavity cancer. Oral Oncol. (2025) 165:107349. doi: 10.1016/j.oraloncology.2025.107349, PMID: 40339433 PMC12893622

[24] Cao F Fang Q Lin R Xu P Zhao Z Jiang K . De-escalated surgery following neoadjuvant chemoimmunotherapy for locally advanced oral squamous cell carcinoma: A retrospective cohort study. Oral Oncol. (2025) 165:107348. doi: 10.1016/j.oraloncology.2025.107348, PMID: 40334311

[25] Qian Y Wu Y . Neoadjuvant chemoimmunotherapy followed by surgery and response- adapted adjuvant radiotherapy for head and neck squamous cell carcinoma. Int J Radiat Oncol Biol Phys. (2024) 120:S155–5. doi: 10.1016/j.ijrobp.2024.07.2173

[26] Seiwert TY Foster CC Blair EA Karrison TG Agrawal N Melotek JM . OPTIMA: a phase II dose and volume de-escalation trial for human papillomavirus-positive oropharyngeal cancer. Ann Oncol. (2019) 30:297–302. doi: 10.1093/annonc/mdy522, PMID: 30481287

[27] Rosenberg AJ Agrawal N Juloori A Cursio J Gooi Z Blair E . Neoadjuvant nivolumab plus chemotherapy followed by response-adaptive therapy for HPV^+^ Oropharyngeal cancer. JAMA Oncol. (2024) 10:923–31. doi: 10.1001/jamaoncol.2024.1530, PMID: 38842838 PMC11157444

[28] Rosenberg AJ Juloori A Jelinek MJ Agrawal N Cursio JF Cipriani N . Neoadjuvant nivolumab plus chemotherapy followed by response-stratified chemoradiation therapy in HPV-negative head and neck cancer. JAMA Oncol. (2025) 11:492–501. doi: 10.1001/jamaoncol.2025.0081, PMID: 40048190 PMC11886870

