# Supplementary material for: Adjuvant radiotherapy for patients with clinical T3–4 oral and oropharyngeal cancer who achieved major pathologic response after neoadjuvant immunochemotherapy and surgery: a propensity score-matched retrospective study
Source: Front Immunol. 2026 Jan 20;16:1681587. doi: 10.3389/fimmu.2025.1681587 (PMC12864408; doi:10.3389/fimmu.2025.1681587)
Supplement: Supplementary Table 1 — Clinicopathologic characteristics in the ypT0–2 cohort. ART, adjuvant radiotherapy; PCR, pathologic complete response; PNI, perineural invasion; LVI, lymphovascular invasion; LNY, lymph node yield; PSM, propensity score matching; ypT, pathologic T stage; ypN, pathologic N stage. [file DataSheet1.pdf]

Supplement Table 1. Clinicopathologic characteristics in the ypT0-2 cohort

Characteristic	Before PSM				After PSM (ypT0-2 cohort)			
	Overall, n=247	non-ART, n=144	ART, n=103	*p*	Overall, n=144	non-ART, n=72	ART, n=72	*p*
Sex, No. (%)				0.739				1
Male	202 (81.8)	119 (82.6)	83 (80.6)		117 (81.2)	59 (81.9)	58 (80.6)	
Female	45 (18.2)	25 (17.4)	20 (19.4)		27 (18.8)	13 (18.1)	14 (19.4)	
Age, years				0.015				0.837
Median (IQR)	54 (46-64)	57 (48-64)	51 (44-60)		53 (45-60)	53 (45-60)	52 (44-59)	
Tobacco use, No. (%)				0.330				0.587
No	171 (69.2)	96 (66.7)	75 (72.8)		100 (69.4)	48 (66.7)	52 (72.2)	
Yes	76 (30.8)	48 (33.3)	28 (27.2)		44 (30.6)	24 (33.3)	20 (27.8)	
Tumor site, No. (%)				0.739				0.812
Oral cavity	185 (74.9)	106 (73.6)	79 (76.7)		109 (75.7)	56 (77.8)	53 (73.6)	
p16- Oropharynx	25 (10.1)	16 (11.1)	9 (8.7)		12 (8.3)	6 (8.3)	8 (11.1)	
p16+ Oropharynx	37 (15.0)	22 (15.3)	15 (14.6)		23 (16.0)	10 (13.9)	11 (15.3)	
Histologic grade, No. (%)				0.301				0.921
Poor	57 (23.1)	31 (21.5)	26 (25.2)		32 (22.2)	16 (22.2)	16 (22.2)	
Moderate	112 (45.3)	62 (43.1)	50 (48.5)		76 (52.8)	39 (54.2)	37 (51.4)	
Well	78 (31.6)	51 (35.4)	27 (26.2)		36 (25.0)	17 (23.6)	19 (26.4)	
cT, No. (%)				0.897				0.402
T3	108 (43.7)	62 (43.1)	46 (44.7)		64 (44.4)	29 (40.3)	35 (48.6)	
T4a	139 (56.3)	82 (56.9)	57 (55.3)		80 (55.6)	43 (59.7)	37 (51.4)	
cN, No. (%)				0.001				0.143
N0	119 (48.2)	80 (55.6)	39 (37.9)		76 (52.8)	40 (55.6)	36 (50.0)	
N1	29 (11.7)	21 (14.6)	8 (7.8)		17 (11.8)	11 (15.3)	6 (8.3)	
N2	95 (38.5)	42 (29.2)	53 (51.5)		48 (33.3)	21 (29.2)	27 (37.5)	
N3	4 (1.6)	1 (0.7)	3 (2.9)		3 (2.1)	0 (0)	3 (4.2)	
ypT, No. (%)				0.284				0.855
T0	168 (68.0)	99 (68.8)	69 (67.0)		97 (67.4)	51 (70.8)	48 (66.7)	
T1	56 (22.7)	33 (22.9)	23 (22.3)		38 (26.4)	17 (23.6)	19 (26.4)	
T2	17 (6.9)	11 (7.6)	6 (5.8)		9 (6.2)	4 (5.6)	5 (6.9)	
T3	3 (1.2)	1 (0.7)	2 (1.9)					
T4	3 (1.2)	0 (0.0)	3 (2.9)					
ypN, No. (%)				< 0.001				0.764
N0	186 (75.3)	127 (88.2)	59 (57.3)		112 (77.8)	57 (79.2)	55 (76.4)	
N1	35 (14.2)	12 (8.3)	23 (22.3)		27 (18.8)	11 (15.3)	14 (19.4)	
N2	24 (9.7)	5 (3.5)	19 (18.4)		5 (3.5)	4 (5.6)	3 (4.2)	

Characteristic	Before PSM				After PSM (ypT0-2 cohort)			
	Overall, n=247	non-ART, n=144	ART, n=103	*p*	Overall, n=144	non-ART, n=72	ART, n=72	*p*
N3	2 (0.8)	0 (0.0)	2 (1.9)					
NICT cycles, No. (%)				0.471				0.597
2 cycles	65 (26.3)	42 (29.2)	23 (22.3)		33 (22.9)	19 (26.4)	14 (19.4)	
3 cycles	158 (64.0)	88 (61.1)	70 (68.0)		93 (63.2)	43 (59.7)	48 (66.7)	
⩾4 cycles	24 (9.7)	14 (9.7)	10 (9.7)		20 (13.9)	10 (13.9)	10 (13.9)	
Pathologic response, No. (%)				0.680				0.719
PCR	169 (68.4)	100 (69.4)	69 (67.0)		97 (67.6)	51 (70.8)	48 (66.7)	
Non-PCR	78 (31.6)	44 (30.6)	34 (33.0)		47 (32.4)	21 (29.2)	24 (33.3)	
PNI, No. (%)				0.164				1
No	242 (98.0)	143 (99.3)	99 (96.1)		148 (100)	74 (100)	74 (100)	
Yes	5 (2.0)	1 (0.7)	4 (3.9)		0	0 (0)	0 (0)	
LVI, No. (%)				0.417				1
No	246 (99.6)	144 (100)	102 (99.0)		148 (100)	74 (100)	74 (100)	
Yes	1 (0.4)	0 (0.0)	1 (1.0)		0	0	0	
LNY, No. (%)				0.216				0.603
≤ 18	78 (31.6)	50 (34.7)	28 (27.2)		52 (36.1)	28 (38.9)	24 (33.3)	
> 18	169 (68.4)	94 (65.3)	75 (72.8)		92 (63.9)	44 (61.1)	48 (66.7)	
Adjuvant therapy, No. (%)								
non-ART	144 (58.3)				72 (50.0)			
PD-1 maintenance	31 (12.6)				18 (12.5)			
ART	103 (41.7)				72 (50.0)			

Abbreviations: ART, adjuvant radiotherapy; PCR, pathologic complete response; PNI, perineural invasion; LVI, lymphovascular invasion; LNY, lymph node yield; PSM, propensity score matching; ypT, pathologic T stage; ypN, pathologic N stage.
